# Physiological Status of Rice Leaf-Roller *Cnaphalocrocis medinalis* (Lepidoptera: Crambidae) Adults Trapped by Sex Pheromone and Floral Odor

**DOI:** 10.3390/insects15090637

**Published:** 2024-08-25

**Authors:** Jianfei Lu, Xiaoming Yao, Ying Shen, Caroline Du, Qianshuang Guo, Yongjun Du

**Affiliations:** 1 Zhejiang Provincial Plant Protection, Quarantine and Pesticide Management Station, Hangzhou 310029, China; ljf86757391@163.com (J.L.); xmyao7@126.com (X.Y.); shenying0227@126.com (Y.S.); 2 Ispxtech Inc., Hangzhou 310018, China; carolined@ispxtech.com; 3 Institute of Pesticide and Environmental Toxicology, Zhejiang University, Hangzhou 310058, China; qianshuang@zju.edu.cn

**Keywords:** *Cnaphalocrocis medinalis*, physiological status, sex pheromone, floral odor, trapping

## Abstract

**Simple Summary:**

The rice leaf-roller *Cnaphalocrocis medinalis* is a migratory pest of rice. Monitoring its population is important in integrated pest management systems. Trapping them with a sex pheromone or plant odor has been used in population monitoring. We studied the physiological status of adults trapped by sex pheromones and floral odors. In the immigrant group, the number of males caught in the floral trap was greater than those caught by the sex pheromone trap. The volumes of testes in adults caught using the above two trapping methods were similar. In the local breeding group, the number of males caught by sex pheromone trapping was greater than that by floral trapping. The volume of testes was smaller in males caught in the floral odor trap compared to the pheromone trap. In the emigrant group, the adult olfactory response to the sex pheromone and floral odor was low. The number of eggs laid by the females in the local breeding group was greater in those caught in the sweep net in comparison with those caught in the floral odor trap.

**Abstract:**

The rice leaf-roller *Cnaphalocrocis medinalis* is an important migratory pest of rice. We conducted a study to determine the physiological status of adults trapped by a sex pheromone and floral odor. In the immigrant group, the number of males trapped by the floral odor was greater than the number caught by sex pheromone trapping. The volume of testes was similar in the above two trapping methods but was smaller than in the sweep net method. The ovary developmental grade, mating rate, and number of matings of females caught in floral odor trap were higher than in those caught in the sweep net. In the local breeding group, the number of males trapped by sex pheromones was greater than the number trapped by the floral odor. The volume of testes was smaller in the floral odor trap compared to the pheromone trap group, with the largest in the sweep net group. The ovarian developmental grade, mating rate, and number of matings of females were significantly higher in the floral odor trap group than in the sweep net group. In the emigrant group, the adult olfactory response to the sex pheromone and floral odor was low. The volume of testes was larger in the sweep net group compared to the moths caught by floral odor trapping. The number of eggs laid by female immigrants trapped by the floral odor and sweep net was similar, while the number in the local breeding group was greater in moths caught with the sweep net in comparison with those caught by the floral odor trap. The difference in egg hatchability between the two trapping methods in both immigrants and local breedings was not significant.

## 1. Introduction

The rice leaf-roller *Cnaphalocrocis medinalis* (Guenée) (Lepidoptera: Crambidae) is an important migratory pest of rice because it reduces rice yield when the infestation is severe [1]. Imprecise and excessive application of chemical pesticides led to the development of pesticide resistance in rice leaf-rollers [2] and even damaged the population of natural enemies [3]. However, adult *C. medinalis* display weak phototaxis. A method called disturbing then moth counting in the early morning has been commonly used in China for pest monitoring, but this method is labor intensive, makes accurate counting hard, and lacks species specificity [4]. Moreover, *C. medinalis* monitoring requires an estimation of its migration status through the dissection of the ovaries [5,6], which is difficult for local extension agents to handle. Therefore, there is an urgent need to develop a simple method with accuracy to monitor this pest. Sex pheromone trapping is conspecific in rice fields but is strongly influenced by the physiological status of the subjects, resulting in reduced accuracy for monitoring [7]. The sex pheromone released by female *C. medinalis* adults has been reported to be a mixture of Z11-18:Ald, Z13-18:Ald, Z11-18:OH, and Z13-18:OH [8,9,10,11,12]. For migratory *C. medinalis*, it is generally believed that the moth needs supplemental nutrition after eclosion, and therefore, floral odor has shown to strongly attract adults [13,14]. There have been many reports on floral odor attracting lepidopteran and dipteran insects [15,16,17,18]. However, because the active compounds based on a floral odor are not as specific as sex pheromones, the attractiveness to non-target pests, beneficial pollinators, and even natural enemies is also significant [19,20]. Therefore, floral odor trapping has been largely used for biodiversity surveys [21]. Whether it is suitable for the monitoring and control of *C. medinalis* remains undetermined.

The olfactory response of insects to sex pheromones and plant odors is closely related to their physiological status [22,23,24,25,26,27], while age is an important factor [27,28]. Male adults that respond with olfactory behaviors to the sex pheromone of conspecific females are sexually mature. For example, the newly emerged male *Agrotis ipsilon* does not respond to the sex pheromone, and its olfactory responsiveness increases with age and sexual maturity [29]. Mating is another important physiological factor [30]. Most migratory species have low and even inconsistent responses to trapping by sex pheromones and floral odors due to their complicated physiological status. *C. medinalis* can mate multiple times during migration [31]. In one study, the developmental level of the ovary in the female moths trapped by the floral odor was relatively high (with grade V) [13]. In the population of local breedings, there were more matings in female moths trapped by the floral odor compared to those caught by the sweep net [13]. The testes of males captured by the sweep net were larger than those trapped by the sex pheromone or floral odor, while the size of those attracted to the sex pheromone was larger than the size of those trapped by the flower odor [13]. However, there is a lack of comprehensive research on the olfactory response of *C. medinalis* moths to the sex pheromone and floral odor associated with migration status. It is also necessary to know the age and mating status of the trapped males and females, as well as the developmental level of the ovaries and fecundity in the female moths. In addition, floral odor has been used for mass trapping of male and female *C. medinalis* in rice fields, but the control potential for the larvae of the next generation is unknown.

In this study, sweep net catching, floral odor trapping, and sex pheromone trapping were used to collect *C. medinalis* adults during the rice-growing season, combined with the dissection of ovaries and testes and laboratory oviposition experiments. The objective was to determine the physiological status, which includes the ovarian development, mating times, and fecundity of female moths, as well as the age of male moths trapped by the sex pheromone and floral odor. 

## 2. Materials and Methods

### 2.1. Cnaphalocrocis Medinalis Trapping and Net Catching in the Paddy Field

The trapping and net-catching experiment was conducted from 1 July to 24 October 2023, in a rice field (29.81N, 121.42E) in Dongwu Town, Ningbo, Zhejiang Province, China. The rice was just transplanted when the experiment started.

The sex pheromone lure consisted of 50 µg of (Z)-11-octadecenal (Z11-18:Ald), 500 µg of (Z)-13-octadecenal (Z13-18:Ald), 90 µg of (Z)-11-octadecen-1-ol (Z11-18:OH), and 120 µg of (Z)-13-octadecen-1-ol (Z13-18:OH) [13]. A polyvinyl chloride (PVC) capillary (100 ± 5 mm length, od. 1.8 mm ± 0.2 mm, id. 0.8 mm ± 0.1 mm) from Newcon Inc. (Ningbo, China) was used as the dispenser.

The floral odor lure contained benzaldehyde, phenylacetaldehyde, phenylethyl alcohol, methyl salicylate, and linalol in an air-permeable black polyethylene (PE) bag (length 86 mm, width 72 mm, and thickness 0.15 mm) as the dispenser (NewCon Inc., Ningbo, China) [13]. The total amount of floral compounds in each dispenser bag was 20 g. The average daily release rate per bag was 0.13–0.15 g [13]. 

An inverted plastic funnel trap (NewCon Inc., Ningbo, China) was used for the experiment [13]. Each trap had a sex pheromone or a floral dispenser. In the experimental paddy field, the bottom of the trap was initially set to about 0.8 m over the soil surface, but when the rice plants were tall enough, the bottom was adjusted to 10–20 cm below the upper surface of the rice plants. The traps were arranged randomly in a straight line, and the distance between neighboring traps was about 30 m. The experiment was replicated 6 times. The distance between replicates was greater than 50 m. The order of traps in each replicate was reversed to reduce the influence of position and wind direction. From 1 July to 24 October, the number of moths in each trap was counted every three days or every day in the morning for dissection, and the pheromone or floral dispenser was replaced once a month.

*Cnaphalocrocis medinalis* adults were captured every day between 07:00 and 09:00 (from 1 July to 24 October) using a sweep net in the rice field. The collected adults were transferred to a mesh cage and brought to the laboratory for dissection.

### 2.2. Cnaphalocrocis Medinalis Rearing in the Laboratory

*Cnaphalocrocis medinalis* larvae were collected from rolled leaves in the rice field and reared with rice leaves in an incubator in the laboratory. The photoperiod in the incubator (Jiangnan, Ningbo, China) was 14:10 h (L:D), and the light was turned off at 18:00. The temperature was 22 ± 1 °C and the humidity was 70% ± 10%. 

Male and female adults were separated immediately after eclosion and placed in different cages that contained 10% sugar water. Male moths were dissected when they were 5 days old. On the day of eclosion, males and females were paired, and during the scotophase, the females finished mating were carefully removed and then used for the oviposition test.

### 2.3. Anatomy of Male and Female Moths

Each *Cnaphalocrocis medinalis* female moth was transferred to a Petri dish containing a 50% alcohol–water solution, and its abdomen was removed under a stereo dissection scope (Motic China Group Co., Ltd., Xiamen, China). The cuticle was torn off with a pair of ophthalmic tweezers in order to expose the ovary and to remove the fat body and other tissues. After the bursa copulatrix was removed, the presence of spermatozoa was used to determine the mating status, and the number of spermatophores was used to determine the mating time. The ovary was photographed, and the grade of ovary development was determined based on the shape and size. The number of eggs in the ovary was recorded as well. The ovarian development was divided into 5 grades according to Zhang et al. [5]. Grade I (transparent and milky white stage): the ovarian tubules were completely transparent or translucent. The fat body was milky white or light yellow and nearly spherical in shape, whereas the mating sac was empty without mating or oviposition. Grade II (yolk deposition stage): half of the egg cells in the middle and lower part of the ovarian tubules were light yellow, indicating the yolk deposition, while the other half remained milky white. The fat body was milky white, plump, round, or oblong. The mating sac was empty without mating or oviposition. Grade III (mature and ready to lay eggs): mature eggs were visible in the ovarian tubules and the ovarian tube plugs but not in the lateral and middle oviducts. The fat body was milky white, plump, and nearly spherical in shape. Only a few female moths were mated; the mating sac was swollen and the spermatophore was visible. Grade IV (peak oviposition stage): ovarian tubules were elongated and contained yellowish mature eggs. There were no ovarian tube plugs, and mature eggs were presented in the lateral and middle oviducts. The fat body was neither full nor filamentous. Mating occurred repeatedly, resulting in a swollen mating sac and multiple spermatophores. Grade V (late oviposition stage): the ovarian tubules were atrophied, and the ends merged into a thin line. A few mature eggs remained in each tubule, often presented in the lateral and middle oviducts. The fat body was sparse and filamentous. The mating sac was swollen and contained multiple spermatophores.

According to the method of Zhang et al. [5], there were three phases in the annual population of *C. medinalis* adults based on the ovary developmental grade, mating status, and the population dynamics. In the immigration phase, less than 2% of the females had reached grade I of ovarian development, and over 95% of the females were mated. In the local breeding phase, 10–35% of the females had reached grade I of ovarian development, and the mating rate was about 70%, whereas in the emigration phase, over 50% of the females had reached grade I of ovarian development and the mating rate was less than 30%. 

Male moths were also dissected under the dissection scope in a Petri dish containing a 50% alcohol–water solution. After the reproductive system was separated from the cuticle and the fat tissue, testes were removed, measured, and photographed as described by Feng et al. [32]. The volume of the testes was calculated as an ellipsoid, and the equatorial radius of the testes was measured using the software Motic Plus Images 2.0. After the length of a, b (along the x and y axes), and the polar radius c were measured, the volume of testes was calculated according to the formula V = 4πabc/3. The imaging equipment used for the female and male moths was a Motic microscope SMZ-168 (MOTIC CHINA GROUP CO., LTD, Xiamen, China) with the photography system Moticam 2506 (Microaudio, Xiamen, China). Male moths at 1, 3, 5, 7, and 9 days old were dissected for the measurement of testes, and 8–9 moths at each of the ages were used as replicates.

### 2.4. Oviposition of Female C. medinalis 

*Cnaphalocrocis medinalis* females collected in the field with sweep nets and floral traps were individually and carefully transferred into 1000 mL plastic bottles with a 50 mL plastic vial containing 10% sugar water, and 3 rice seedlings grown in a small pot filled with soil mix were placed in the bottle. The moths stayed in the bottle until the oviposition was completed at a temperature of 25 ± 1 °C, a humidity of 60 ± 5%, and a photoperiod of 14L:10D. They can survive for 1–7 days until oviposition depending on the source of female moths. The number of eggs laid and the number of eggs hatched were recorded. 

### 2.5. Statistical Analysis

SPSS version 17 [33] was used to analyze the data in this study. The resulting data of *C. medinalis* adults were analyzed using one-way ANOVA. Pairs of treatment means were compared and separated using Duncan’s multiple range test. The number of *C. medinalis* adults from trap-collected samples was log(y + 1)-transformed prior to the ANOVA in order to normalize the data [34]. Proportions were compared using the chi-square test. The Pearson correlation method was used to determine the relationship between the volume of testes and the age of male moths.

## 3. Results

### 3.1. Cnaphalocrocis Medinalis Moths Captured by Sex Pheromone, Floral Odor, and Sweep Net 

**Figure 1:**
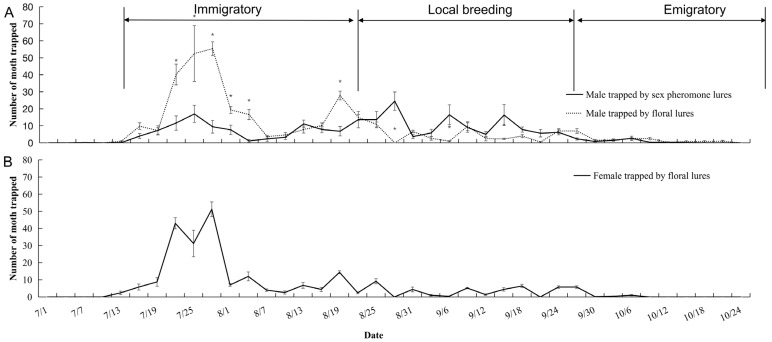
Seasonal population dynamics and migration status of *C. medinalis* derived from floral odor and sex pheromone trapping: (**A**) males trapped by the sex pheromone and floral odor; (**B**) females trapped by the floral odor. * Indicates statistically significant difference between treatments in the same sampling date (*p* < 0.05).

Based on the criteria in Zhang et al. [5] for ovary developmental grade and mating status, there were three phases in the annual population of *C. medinalis* adults captured with the sweep net in the patty field: immigrants (from 13 July to 15 August), local breedings (from 21 August to 24 September), and emigrates (from 27 September to 22 October) (Figure 1). During the first phase (immigrants), two peaks of *C. medinalis* males were detected from mid-July to late August in the floral odor trap, during which the highest number (55.3 ± 4.1/trap) was on 25 July, followed by (27.8 ± 2.6/trap) 19 August (Figure 1A). The number of males caught from 24 July to 4 August in the sex pheromone trap was lower than that in the floral odor trap (*t* = 4.48, *df* = 94, *p* < 0.001) (Figure 1A). On August 19, the number of males caught in the sex pheromone trap was also significantly lower than that in the floral odor trap (*t* = −5.01, *df* = 10, *p* < 0.001). During the second phase (local breedings), the mean number of males in the sex pheromone trap in 3 days was 10.4 ± 1.4, which was significantly higher than that of male moths trapped by the floral odor, which was 4.3 ± 0.6 (*t* = −4.11, *df* = 130, *p* < 0.001). During the third phase (emigrants), after September 30, the number of males caught in the sex pheromone and floral odor traps was very low (Figure 1A). The population dynamics of females caught in the floral odor trap was similar to that of males (Figure 1B). The sex ratio of females to males caught in the floral odor trap was 1.39.

### 3.2. Anatomy of C. medinalis Adults Captured by Sex Pheromone, Floral Odor, and Sweep Net 

**Figure 2:**
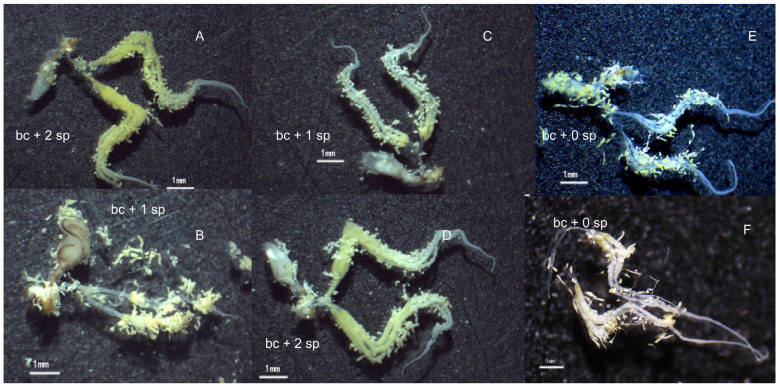
*C. medinalis* female ovaries in different migration phases: (**A**) immigrants caught in sweep net; (**B**) immigrants caught by floral trapping; (**C**) local breedings caught in sweeping net; (**D**) local breedings caught by floral trapping; (**E**) Grade I of emigrates caught in sweep net; (**F**) Grade II of emigrates caught in sweep net. Bc: bursa copulatrix; sp: spermatophore; the number before sp means the mating times.

The dissection of the reproductive systems of *C. medinalis* females in the three phases of migration revealed differences in the developmental levels of ovaries and mating times (Table 1 and Figure 2). In the immigrant group, the number of mated females was 12.8% greater in the floral odor trap than in the sweep net (*p* < 0.05), and in those mated, the mean mating time was 1.5 in the floral odor trap versus 1.1 in the sweep net (*p* < 0.05). The ovarian developmental level was over 30% more advanced in the floral odor trap (*p* < 0.05), but egg numbers in the ovaries were similar to those in the two trapping methods (*p* > 0.05). In the local breeding group, the mated females, mating times, and ovarian developmental levels were 4.0-, 2.8-, and 1.6-fold higher in the floral odor trap compared to those in the sweep net, respectively (*p* < 0.05). However, the egg number in the ovary was 26% less in the floral odor trap. For the emigrants in the sweep net, the percentage of mated females was less than 10%, and in those mated, the mean mating time was one, the mean ovarian developmental grade was close to II, and the mean number of eggs in the ovary was 328. However, the emigrants were not attracted to the floral odor trap.

**Figure 3:**
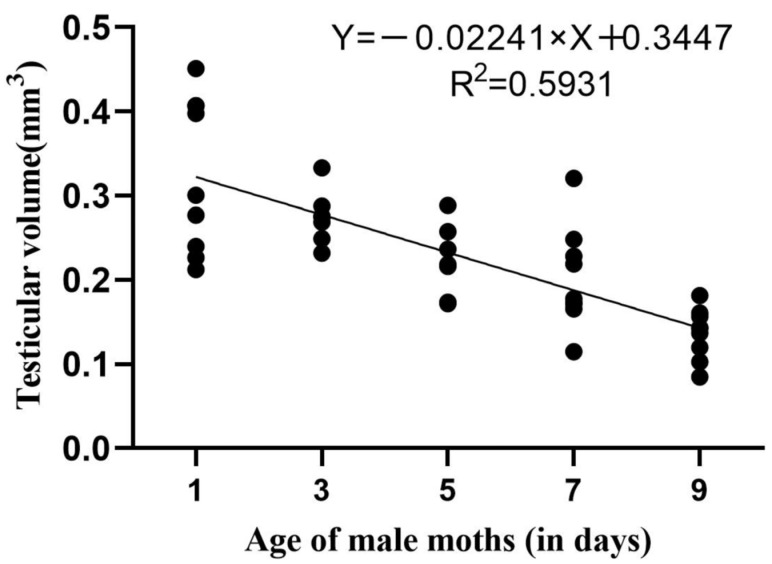
Size of *C. medinalis* male testes in the three migration phases and the effect of age on the testicular volume.

Volumes of male testes from moths in the three migration phases collected from the sweep net, floral odor, and sex pheromone traps are shown in Table 2 and Figure 3. In the immigrant group, the volume of male testes by floral odor trapping was 62% smaller and the volume by sex pheromone trapping was 15% smaller compared to that by net catching (*p* < 0.05). In the local breeding group, the volume of male testes was 65% and 30% smaller in the floral odor trap and the sex pheromone trap, respectively, than that in the sweep net (*p* < 0.05). In the emigrant group, the volume of male testes was 48% smaller in the floral odor trap in comparison with that in the sweep net (*p* < 0.05). 

The average volume of testes in the 1-, 3-, 5-, 7-, and 9-day-old *C. medinalis* male moths was 0.32 ± 0.03, 0.27 ± 0.01, 0.22 ± 0.01, 0.20 ± 0.02, and 0.14 ± 0.01 mm^3^, respectively. Testes volume and age were negatively linearly correlated (*R* = −0.7713, *p* < 0.01) (Figure 3).

According to the functional relationship in Figure 3, the average age of male moths captured by the sweep net in the local breeding group was 6.4 ± 0.5 days, that of males caught by sex pheromone trapping was 9.3 ± 0.4 days, and that of males caught by floral trapping was 11.9 ± 0.4 days. Male moths collected in the immigrant group were relatively older (Table 2).

### 3.3. Oviposition and Egg Hatching Rate by C. medinalis Females in Different Migration Phases

**Figure 4:**
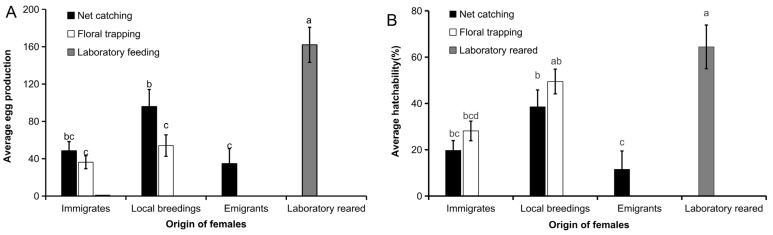
*C. medinalis* oviposition and egg hatchability in different migration statuses: (**A**) eggs oviposited by the females; (**B**) egg hatchability. Bars in the same figure with different letters are significantly different at *p* < 0.05.

The oviposition and egg hatching rate in *C. medinalis* females captured by floral odor and sweep net trapping in the three migration phases are shown in Figure 4. In the first phase (immigrants), the number of eggs laid by the females was not significantly different between the two capture methods (*p* > 0.05), but in the second phase (local breedings) it was 43.6% less in the floral odor trap (*p* < 0.05). In each of the first two phases (immigrants and local breedings), egg hatching rates were similar in both the floral odor trap and the sweep net groups (*p* > 0.05). 

The results showed that there were significant differences in the number of eggs laid by females in different migration phases (*F* = 7.26, *df* = 172, *p* < 0.001). The number of eggs laid by the laboratory-reared females was the highest, 162.0 ± 18.8, which was significantly higher than those of field-collected females in the three migration phases. Among the female moths caught with the sweep net, the number of eggs laid by local breeding females was 95.9 ± 18.3, which was significantly higher than the 34.6 ± 16.2 eggs of the migratory females caught by the sweep net. The number of eggs laid by the local breeding females caught by the floral trap was 54.1 ± 11.5, which was significantly lower than that of the local breeding female moths caught by the sweep net, and the number of eggs laid by the immigrant population caught by the floral trap was 36.2 ± 7.0 (Figure 4A). The hatching rates of eggs laid by the females of different physiological statuses were also significantly difference (*F* = 6.67, *df* = 172, *p* < 0.001). The hatching rate of eggs laid by laboratory-reared females was the highest at 64.4 ± 9.4%. The hatching rate of eggs laid by net-caught local breedings was 38.6 ± 7.2%, that of eggs of immigrant females was 19.7.0 ± 4.2%, and that of eggs of emigrant females was 11.5 ± 9.4%. The hatching rate of eggs laid by immigrant females in the floral trap group was 28.1 ± 4.2%, and the hatching rate of eggs laid by local breeding females in the floral trap group was 49.5 ± 5.3% (Figure 4B).

## 4. Discussion

This study tested capturing *C. medinalis* moths by using sex pheromone and floral odor traps in different migratory phases. Both the sex pheromone and floral odor mediate *C. medinalis* adult mating behavior through the olfactory system. However, adult age [28], physiological status [35,36], the nutritional status of larvae [37,38,39], adult feeding [40], and biotic and abiotic factors in the environment [41,42] have a great impact on the olfactory response of insects. The behavior of adult *C. medinalis* in different migration statuses is influenced by a combination of these factors. Therefore, the behavioral regulation by floral odor and sex pheromones is closely related to the migration status of male and female *C. medinalis*. Our results demonstrated that the number of immigrants trapped by the floral odor was greater than that by the sex pheromone, which is obviously related to their physiological status. The number of males captured by the sex pheromone was relatively small, suggesting that a substantial number of males had completed their first mating during migration. This is consistent with a previous report that 60% of the ovaries of female *C. medinalis* trapped by high-altitude searchlights were mature and mated [43]. Although it is generally believed that insects require nutritional supplementation after emergence [44], the reality is that insects require more water after emergence [45]. The ovarian development level of *C. medinalis* adults fed only with water was significantly lower than that of females provided with supplementary nutrition [46]. Migratory insects consume a lot of energy during the process of multiple takeoffs and landings, so the energy reservation of *C. medinalis* adults is related to their migratory status. The emigrants store more fat in their body than the local breedings, while the immigrants have the lowest fat content [47]. Therefore, this may be the reason why the number of immigrants in the floral trap was relatively large. However, the results of this study confirmed that female adults trapped by the floral odor have already mated and laid eggs, and the testes of the males trapped are relatively smaller.

The priority for herbivorous insects after emergence is to search for their conspecific partner to mate, find a suitable habitat and host for the next generation, and finally lay eggs. Based on the level of ovary development, the mating rate of females, and the population size, the *C. medinalis* population was divided into three migration phases: immigrants, local breedings, and emigrants [48]. Because the migration of *C. medinalis* adults occurs in the early stage of reproduction, and migrating *C. medinalis* moths tend to have a longer reproductive preparation and the pre-oviposition period, the mating rate and fecundity of females are reduced [49]. The lifespan of *C. medinalis* adults is long, and the time interval between matings is longer [50]. Therefore, the population of immigrants may have mated during the migration, and there will be a certain time period prior to the next mating [51,52]. After the mating quiescent period, the calling behavior will be initiated again [51,52]. The mating behavior of *C. medinalis* has been studied in detail [53,54]. Adult *C. medinalis* can mate 1–5 times, but usually 1–3 times [13,31]. Multiple matings can increase egg production and the egg hatching rate [31], but the increase in mating age reduces the reproductive capacity [31]. This study showed that there was a reduction in egg production and egg hatchability of females caught in the floral trap in the immigrant and local breeding groups. However, female *C. medinalis* emigrants have a low ovarian grade level and are sexually immature prior to migration. Our results also showed that the corresponding male moths were not sensitive to the female sex pheromone. Thus, the emigrants had olfactory behavioral responses to neither the sex pheromone nor the floral odor.

This study confirmed that there were differences in sexual maturity among *C. medinalis* adults caught by the sex pheromone, floral trapping, and sweep net in the paddy field. In the immigrant group, 100% of the females captured in the floral trap had already mated, while only 87.2% of those captured by the sweep net had mated. Adult males caught in the sex pheromone trap were sexually mature and looking for a mate. In the immigrant and the local breeding groups, although the testes of male moths caught in the sex pheromone trap were smaller than in those captured by the sweep net, they were larger than in those captured in the floral odor trap. It can be inferred that the males caught by sex pheromone trapping were mated once. Based on the functional relationship between age and the volume of testes, it was estimated that the males caught by floral trapping were already about 13 days old in the immigrant group and 12 days old in the local breeding group. The ovary level, mating rate, and number of matings of the females in the floral trap group were all higher than those captured in the sweep net, but there was no significant difference in the number of eggs in the dissected ovaries, which was only about 50% of the eggs of normally emerged female moths. Females in the immigrant group caught in the floral trap were older, had a smaller number of eggs, and had lower egg hatchability. At the same time, the number of males trapped by the female sex pheromone in the immigrant group was less than 50% of that trapped by floral odor. However, in the local breeding group, the number of males attracted by the sex pheromone was over 50% greater than that trapped by the floral odor. In the emigrant group, the number of males trapped by both the sex pheromone and floral odor was extremely low. Therefore, the combination of the sex pheromone and floral trapping can determine the migration status of *C. medinalis*. 

In the rice production region of China, the conventional method used to monitor the population dynamics and the migration phase of *C. medinalis* is labor intensive and inaccurate. Researchers have to work in the early mornings to count the number of flying moths immediately after disturbing rice plants in a patty field and then collect and dissect the females to grade the ovary development [4,5,6]. Our study on the physiological status of *C. medinalis* adults caught by sex pheromone and floral odor trapping can help simplify population monitoring. The male adults trapped by the sex pheromone were sexually mature and in the status of searching for mates. Therefore, if half of the males trapped by the floral odor is greater than the number trapped by the sex pheromone, this population should be considered as the immigrants. Otherwise, they should be considered as the local breedings. If there are few adults trapped by both the sex pheromone and floral odor, and the population in the field was large, they should be considered as the emigrants.

In summary, in the immigrant group, the number of *C. medinalis* males trapped by the sex pheromone was less than half of that trapped by floral trapping, and the age of males in the pheromone trap was 2–3 days younger. In the local breeding group, the number of males trapped by the sex pheromone was more than half of that by the floral odor, and the proportion of males unmated was also greater by sex pheromone trapping. The females had higher developmental grade of ovaries in the floral odor trap group compared to the group caught with the sweep net. In the floral odor trap group, all the female immigrants had mated, while the mating rate in the local breeding group exceeded 92%. The number of eggs laid in the local breeding group was low. The attractiveness of the sex pheromone and the floral odor to the emigrants was poor in the field. The combination of sex pheromone and floral trapping can not only monitor the population dynamics of *C. medinalis* moths but can also indicate their migration phase. 

## Figures and Tables

**Table 1:** Anatomical data of *C. medinalis* females in their different migration phases.

Migration	Trapping Methods	Total No. of Females Dissected	% of Females Mated	Mating Times	Ovarian Developmental Grade	No. of Eggs in the Ovary
Immigrants	Floral trapping	19	100.0 ± 0.0 a	1.5 ± 0.2 a	4.7 ± 0.1 a	199.8 ± 21.7 a
	Net catching	47	77.8 ± 14.7 b	1.1 ± 0.1 b	3.6 ± 0.1 b	225.1 ± 15.0 a
Local breedings	Floral trapping	38	95.0 ± 5.0 a	1.1 ± 0.1 a	3.6 ± 0.1 a	209.8 ± 12.2 b
	Net catching	60	17.3 ± 6.4 b	0.4 ± 0.1 b	2.2 ± 0.2 b	282.6 ± 9.7 a
Emigrants	Floral trapping					
	Net catching	22	14.0 ± 9.8	1.0 ± 0.0	1.7 ± 0.2	328.0 ± 27.9
	Collecting pupae in the field					397.3 ± 21.4

Numbers in the same column within the same migration status followed by different letters are significantly different at *p* < 0.05.

**Table 2:** Anatomical data of *C. medinalis* males in their different migration phases.

Migration Phase	Trapping Methods	Total No. of Dissected Males	Volume of Testes (mm^3^)	Calculated Age (d)
Immigrants	Sex pheromone trapping	36	0.11 ± 0.00 b	10.4 ± 0.3
	Floral trapping	19	0.05 ± 0.01 b	13.1 ± 0.4
	Net catching	17	0.13 ± 0.02 a	9.5 ± 0.8
Local breedings	Sex pheromone trapping	23	0.14 ± 0.01 b	9.3 ± 0.4
	Floral trapping	32	0.07 ± 0.01 c	11.9 ± 0.4
	Net catching	32	0.20 ± 0.01 a	6.4 ± 0.5
Emigrants	Floral trapping	5	0.11 ± 0.02 b	10.1 ± 0.7
	Net catching	5	0.21 ± 0.03 a	5.9 ± 1.2

Numbers in the same column within the same migration status followed by different letters are significantly different at *p* < 0.05.

## Data Availability

The data that support the findings of this study are available upon reasonable request.
